# Hypothalamic Microglial Heterogeneity and Signature under High Fat Diet–Induced Inflammation

**DOI:** 10.3390/ijms22052256

**Published:** 2021-02-24

**Authors:** Natália Ferreira Mendes, Carlos Poblete Jara, Ariane Maria Zanesco, Eliana Pereira de Araújo

**Affiliations:** 1 Faculty of Nursing, University of Campinas, Campinas 13083-887, Brazil; natalia.mendesss@gmail.com (N.F.M.); blinkeado@gmail.com (C.P.J.); 2 Laboratory of Cell Signaling, Obesity and Comorbidities Research Center, University of Campinas, Campinas 13083-864, Brazil; arianemariazanesco@gmail.com; 3 School of Medical Sciences, University of Campinas, Campinas 13083-887, Brazil

**Keywords:** microglia, gliosis, hypothalamus, high-fat diet, obesity, transcriptomic

## Abstract

Under high-fat feeding, the hypothalamus atypically undergoes pro-inflammatory signaling activation. Recent data from transcriptomic analysis of microglia from rodents and humans has allowed the identification of several microglial subpopulations throughout the brain. Numerous studies have clarified the roles of these cells in hypothalamic inflammation, but how each microglial subset plays its functions upon inflammatory stimuli remains unexplored. Fortunately, these data unveiling microglial heterogeneity have triggered the development of novel experimental models for studying the roles and characteristics of each microglial subtype. In this review, we explore microglial heterogeneity in the hypothalamus and their crosstalk with astrocytes under high fat diet–induced inflammation. We present novel currently available ex vivo and in vivo experimental models that can be useful when designing a new research project in this field of study. Last, we examine the transcriptomic data already published to identify how the hypothalamic microglial signature changes upon short-term and prolonged high-fat feeding.

## 1. Introduction

Hypothalamic inflammation is a condition frequently observed in experimental models of diet-induced obesity (DIO) [1,2,3] and obese humans [4,5,6]. This inflammatory response is mainly triggered by excessive saturated fatty acids (SFAs) from the diet [7,8,9], which reach the neural tissue mainly through the median eminence (ME), where fenestrated vascular endothelium lacks a blood–brain barrier (BBB) [10,11]. Brain perivascular macrophages (PVMs) also react to excessive free fatty acids (FFAs) circulating in the blood vessels, with a consequent increase in BBB permeability [12,13]. Glial cells, such as astrocytes and microglia, quickly sense and react to the presence of those SFAs in the hypothalamic parenchyma, releasing pro-inflammatory cytokines, chemokines, and reactive oxygen species (ROS) [14,15]. If the stimulus persists, the hypothalamic neuronal network may be damaged, resulting in neuro-inflammation, which eventually leads to energy balance disruption [16,17], and finally, to neuronal dysfunction/apoptosis [18].

Researchers have shown that hypothalamic inflammation initiates just a few hours/days upon high-fat feeding [5,15,19]. After the onset of the inflammatory response, bone marrow–derived cells (BMDC) and other peripheral immune cells, such as neutrophils, lymphocytes, and regulatory T (Treg) cells can arise into the hypothalamic parenchyma in a time-dependent manner, directly affecting glial functions [12,20,21]. To avoid further metabolic complications, hypothalamic neuronal and non-neuronal cells, along with peripheral immune cells, should act together in an orchestrated mode beginning with the earliest phase of the inflammatory response.

The molecular mechanisms underlying microglial immune and metabolic interactions with other cell types under high-fat diet (HFD)-induced hypothalamic inflammation still require a more detailed exploration [22]. Recent data unveiling microglial diversity and signatures throughout the brain have contributed to the development of novel state-of-the-art approaches in experimental studies, which can be valuable for this field in the coming years.

In this review, we explore novel findings of hypothalamic microglial diversity from rodents and humans. We examine the subtypes of microglia that may be involved in HFD-induced hypothalamic inflammation and investigate how these cells interact with astrocytes upon high-fat feeding. Beyond that, we discuss which models can be useful to get the most reliable data when studying distinct subsets of microglia, myeloid cells, and border-associated macrophages (BAMs). Last, we investigate novel transcriptomic data already published to clarify how the hypothalamic microglia signature changes under saturated fat consumption.

## 2. Microglial Heterogeneity in the Hypothalamus

Microglia were identified by Pío del Río-Hortega in 1919, but only in the last two decades has the interest in these cells grown exponentially, with the discovery of their unique origin in the yolk sac and motile capacity [23,24]. Since their identification, microglia have been studied as a unique macrophage-like cell type in the central nervous system (CNS), able to quickly react to a wide range of stimuli by switching their phenotype activation between M1 and M2 subtypes [25,26]. According to this past view, microglia represent a naïve cell type that could equally react to any stimuli by acquiring a predetermined phenotype.

Most recently, advances in genetic tools have been developed and extensively employed in experimental research, enabling a deeper understanding of microglial diversity. Stratoulias et al. [27] recently proposed a new classification, in which microglia constitute a heterogeneous cell group, and each subtype has distinct properties and physiological functions, reacting differently to stimuli. This new view is based on the regional steady-state heterogeneity of microglia and their broad gene marker diversity. Curiously, from six putative microglial subtypes with unique specializations presented by these authors, the only one found in the hypothalamic area is dark microglia (DM). Despite their classification in subtypes, it is important to highlight the existence of many other types of microglia showing distinct features and functions in the hypothalamic area, which were not considered, or at least did not show up, in their categorization.

DM are also found in the hippocampus, cerebral cortex, and amygdala [28]. These cells can only be visualized through high-spatial-resolution transmission electron microscopy and display markers of oxidative stress, such as a condensed, electron-dense cytoplasm and nucleoplasm, dilatation of the Golgi apparatus and endoplasmic reticulum, mitochondrial alteration, and a partial to complete loss of the heterochromatin pattern. Functionally, DM are very active and show extremely thin processes, which allow them to make contact with synaptic elements [28]. Despite being rarely observed in healthy young adult mice, DM are widespread upon chronic stress, ageing, in CX3C chemokine receptor 1 (CX3CR1)-knockout (KO) mice, and in Alzheimer’s disease pathology (APP/PS1 model) [27].

It remains unknown through which mechanisms DM are involved in the central inflammatory response, but it is reasonable to speculate that DM functions, similarly to other microglial subtypes, are influenced by peripheral signals. Savage et al. [29] showed that 24 h after an acute systemic injection of lipopolysaccharide (LPS), there are alterations in the inflammatory profile and in the microglial ultrastructure in the hippocampus, with no direct impact on DM. Curiously, DM are largely found in C-C chemokine receptor type 2 (CCR2-KO) mice, which present impaired recruitment of peripheral monocytes to the brain [30]. This finding is particularly interesting because upon prolonged HFD consumption, these peripheral cells are recruited to the hypothalamic parenchyma, as previously mentioned, similarly to what happens in many types of acute CNS injury [21].

Since their first description in 2016 [28], studies have described several DM features in rodents [31,32,33,34,35,36] and humans [37]. However, none of these studies have focused on DM roles specifically in the hypothalamus. Advances in this subject remain necessary to clarify how DM crosstalk with other microglia under hypothalamic inflammation, whether DM are involved in this recruitment of immune cells to the brain, and which peripheral stimuli can affect hypothalamic DM functions.

Microglia simultaneously express several hallmarks, such *as Iba1, Cx3cr1, P2ry12, Tmem119, Trem2, Cd11b, Hexb, Csf1r, Itgam,* and *Siglec*, among others [38,39,40,41,42,43]. However, there is no unique expression pattern of these transcripts in these cells, that is, it varies according to the pathological condition, age, sex, species, and brain area [27]. Particularly in mice, Valdearcos et al. [20] recently showed through immunofluorescence assays that the microglial signature varies according to hypothalamic nuclei and the dietary fat content. Thus, at least in mice, some CX3CR1+ cells in the arcuate nucleus (ARC) overlap with Tmem119+ or P2ry12+ cells, while other CX3CR1+ cells do not. When gliosis occurs upon consuming a HFD, ionized calcium binding adaptor molecule 1 (Iba1+) cells become widespread in the ME, ARC, and ventromedial nucleus (VMH), while Tmem119+ and P2ry12+ cells remain more restricted to the VMH when compared with the brain slices obtained from mice fed on chow diet. Some studies involving DM also show this heterogeneity, because these cells barely express *Iba1*, *Cx3cr1*, and *P2ry12*, but robustly express cluster of differentiation molecule 11B (*Cd11b*), which is involved in their synaptic pruning role [44,45].

These data indicate that some hypothalamic microglial subsets may have anti-inflammatory functions, depending on the transcriptomic profile of each subtype and their status (steady-state or reactive). It is plausible to consider that it may also happen with other hallmarks, which explains why using only one surface marker for microglial staining, choosing a single cell line for ex vivo experiments, or even examining a single transgenic mouse model for manipulating microglia implicates a methodology bias.

Disease-associated microglia (DAM) are another subset of CNS resident macrophages that have been recently identified in experimental models of neurodegeneration and demyelination [46]. These cells are characterized by the expression of several genes, such as *Apoe*, *Clec7a*, *Cst7*, and *Spp1*. They are mainly found at sites of neurodegeneration and might play a protective role [47]. Interestingly, DAM hallmarks are also observed in human Alzheimer’s disease post-mortem brains [46,48]. Triggering receptor expressed on myeloid cells-2 (TREM2) is also highly expressed by DAM; the activation of its intracellular signaling is essential for the transition of homeostatic microglia to the DAM state [49]. Both Toll-like receptor 4 (TLR4) and TREM2 can recognize different pathogen-associated molecular patterns [50,51] and other ligands, such as gram-negative bacteria [52], lipids [53], apolipoproteins (ApoE, ApoJ, and ApoA) [54,55,56], and nucleic acids released by apoptotic cells [57]. Because TREM2 and TLR4 share some ligands, it is not easy to identify which signaling pathway is activated through each receptor by these stimuli. However, experiments regarding TREM2 inhibition in combination with TLR4 stimulation by LPS have started to clarify the involvement of each receptor in neuro-inflammation [58].

An important characteristic observed in HFD-induced hypothalamic inflammation is the activation of nuclear factor kappa-light-chain-enhancer of activated B cells (NF-κB) [14,59,60,61,62]. Conversely, inhibition of IKKβ/NF-κB signaling in microglia expressing CX3CR1 ameliorates DIO and hypothalamic inflammation [20]. In the CNS, TREM2 is widely expressed in microglia [63,64], where it acts by negatively regulating the activation of NF-κB [65]. In BV2 cell culture, TREM2 overexpression inhibits PI3K/AKT and NF-κB signaling pathways [66,67]. Interestingly, LPS reduces the expression of TREM2 in these cells through the activation of JNK and NF-κB, resulting in a vicious cycle [66]. Recently, Zhang et al. [68] treated BV2 cells with curcumin, a bioactive compound isolated from *Curcuma longa* with anti-inflammatory and antioxidant activities, and LPS; they observed reduced microglial activation via TLR4/NF-κB when compared with microglial cells treated only with LPS, which occurred in parallel to the increased TREM2 expression, reinforcing the potential anti-inflammatory role of this receptor.

**Figure 1:**
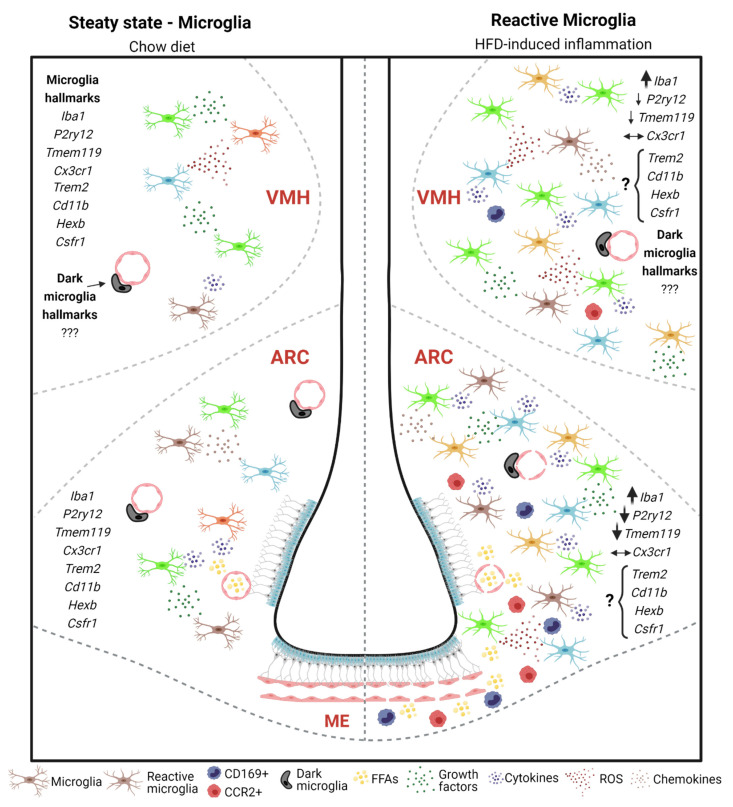
Microglial heterogeneity in the hypothalamus of rodents fed on chow or a high-fat diet (HFD). At steady state, microglia can be targeted by several hallmarks and are widely distributed in the hypothalamic parenchyma. Dark microglia (DM) are only visualized by transmission electron microscopy and are close to blood vessels and neuronal synapses. Hallmarks of DM are well known in hippocampus, but remains barely explored in the hypothalamus. In HFD-induced hypothalamic inflammation, reactive gliosis is observed and microglia change their spatial distribution and molecular signature. Microglia cells react by increasing the release of pro-inflammatory cytokines, reactive oxygen species (ROS), and growth factors. In the ARC, ME, VMH, there is a huge increase in Iba1+ cells (wide arrow) and a decrease in P2ry12+ and Tmem119+ cells, which become more restricted to the VMH (thinner arrows when compared to their arrows in the ARC). How other microglial hallmarks (e.g., *Trem2*, *Cd11b*, *Hexb*, *Csfr1*, among others) behave under HFD have not been studied yet in this inflammatory process. The role of DM and changes in their hallmarks in this specific inflammatory response have still not been explored. A leaky blood–brain barrier (BBB) allows free fatty acids (FFAs) accumulation in the hypothalamic parenchyma, boosting inflammation. If HFD persists for some weeks, peripheral myeloid cells, such as CD169+ and CCR2+ cells, are chemoattracted and infiltrate the hypothalamic parenchyma, but their functions in the HFD-induced inflammation need to be further studied. Abbreviations: ARC, arcuate nucleus of the hypothalamus; VMH, ventromedial nucleus of the hypothalamus; ME, median eminence.

Microglial dynamics and density vary between brain areas and LPS doses during infection-induced inflammation [69]. In addition, considering that both LPS and SFAs can activate TLR4 and TREM2 intracellular signaling pathways, it is reasonable to speculate that a HFD could also have some effect on these microglial characteristics, especially in the hypothalamus. Yet, whether continued activation of TREM2+ cells under prolonged HFD-induced inflammation is implicated in the development of neurodegenerative diseases remains unexplored. The broad diversity of hypothalamic microglia at steady state and during the HFD-induced inflammatory response is shown in Figure 1.

Most transcriptomic data currently available about microglia heterogeneity is from studies with rodents [39,70,71,72]. By contrast, human microglial diversity has only begun to be comprehended in the last few years. Masuda et al. [39] recently identified several clusters of microglia in healthy human brains and in the brains of patients with multiple sclerosis, but these authors evaluated only the cortex and temporal lobe. Similarly, Sankowski et al. [73] identified several microglial subsets in the human brain during homeostasis and some diseases through the combination of two high-dimensional technologies, single-cell RNA sequencing (scRNA-seq) and multiplexed mass cytometry (CyTOF), although the hypothalamus was not included in the analysis. Likewise, Böttcher et al. [74] applied multiplexed CyTOF to detect microglia regional heterogeneity in human post-mortem samples from the subventricular zone, thalamus, cerebellum, temporal lobe, and frontal lobe.

Unfortunately, there is still a lack of data from hypothalamic human microglia in the hypothalamus because there are several methodological difficulties in collecting human brain samples and keeping them cryopreserved without damage. These issues have limited large-scale studies. In addition, while recent advances in transcriptomics, multiplex protein expression analysis, and methods to detect chromatin structure have revealed many facets and details about microglia in rodents and humans, the correlation between the genome-related information and their features remains to be explored.

## 3. Hypothalamic Microglia–Astrocyte Crosstalk

CNS homeostasis, development, injury, and repair are precisely controlled by appropriate cell–cell communication. Nevertheless, studies investigating microglial functions have been focused on microglia isolation, neglecting that physiological actions of these cells are part of a complex network involving other cell types. Therefore, new tools have been widely applied in experimental studies, allowing the determination of an infinite number of intercellular interactions by connecting ligands to target genes across cell types and tissues [75,76,77]. Unsurprisingly, the inflammatory process in the hypothalamus, observed in obesity, drives multiple harmful outcomes that can affect the interactions between microglia and other cell types [78,79]. As astrocytes and microglia play active roles from the onset of the hypothalamic inflammatory response, here we focus on their crosstalk under this process.

While microglia comprise only 5% to 10% of the total number of CNS cells in humans and rodents [80], astrocytes are the largest cell component of the brain, comprising at least 50% of all CNS cells [81]. Astrocytes make intimate contacts with synapses, blood vessels, and other glial cells, thus controlling synaptic transmission, BBB structure and function, and sensing nutrients and hormones in the blood [82,83]. They are also involved in the regulation of lipid metabolism and storage [84] and some studies have been indicating that astrocytes can also express some hormone receptors, such as leptin, insulin, ghrelin, and glucocorticoid receptors [85,86,87,88].

Coordinated crosstalk between glial cells is a determinant in homeostatic and pathological conditions [89]. Microglia and astrocytes interact via contact-dependent and secreted factors, such as growth factors, neurotransmitters, cytokines, chemokines, innate-immunity mediators, mitogenic factors, ROS, and metabolic mediators such as glutamate, which can be used for cell metabolism and may also mediate tissue changes [90]. These glial cells play a crucial role in synapse development and function in the healthy CNS, forming the ‘quad-partite’ synapse [91], which is essential for neuro-immune communication [90], critically contributing to brain homeostasis [92].

Both microglia and astrocytes quickly become activated upon an injury or any inflammatory stimulus [93]. Reactive microglia release several pro-inflammatory cytokines that induce astrocytic activation [75,93]. Likewise, HFD-induced microglial activation results in astrocytic proliferation, morphological changes, and increased production of cytokines and growth factors [61,84,94,95,96]. Classically, these morphologic and functional adaptations by glial cells under inflammation are known as gliosis [5,97] and are usually described in rodents fed on HFD for weeks [20,98], or even after very short periods of high-fat feeding [15].

In the hypothalamus, all these astrocytic characteristics are essential for controlling energy homeostasis. NF-κB signaling inhibition by IKKβ deletion in astrocytes (GFAP-Cre mice) reduces HFD-induced hypothalamic inflammation and reactive astrogliosis and attenuates DIO and glucose intolerance [14].

Similarly, knockdown of TGF-β1—which is predominantly synthesized by astrocytes, specifically in the ARC of HFD-fed mice—reduces TGF-β/SMAD and NF-κB signaling pathways and, consequently, also attenuates the inflammation [99]. Interestingly, this approach of hypothalamic TGF-β1 knockdown presents many metabolic benefits, preventing obesity development even under HFD. Countless microglial functions depend on TGF-β1, including their maturation and activation, both in homeostatic conditions and in response to any inflammatory stimuli [100,101]. In addition, the accurate functioning of the TGF-β1 signaling pathway is crucial to prevent excessive microglia activation [102,103].

In astrocyte cell culture, treatment with palmitic acid, an SFA largely found in HFD, evokes lipid droplet accumulation [95], culminating in an inflammatory response, characterized by an increased production of chemokines, such as chemokine C-C motif ligand-2 (CCL2), also called monocyte chemoattractant protein-1 (MCP-1). Curiously, the main chemotactic mechanism described in the hypothalamic inflammation is mediated by fractalkine (CX3CL1), a chemokine produced by hypothalamic neurons that acts through the fractalkine receptor (CX3CR1) [104]. However, another possible chemokine involved in the peripheral recruitment of immune cells to the hypothalamus is MCP-1 [105]. Peripheral immune CCR2+ and CD169+ cells arise in the hypothalamic parenchyma upon chronic periods of HFD [12,20]. CCR2+ cells of obese mice can enter into their white adipose tissue, and this chemo-attraction is mediated by the MCP-1/CCR2 axis [106].

In the CNS, the MCP-1/CCR2 axis has also been presented as an important chemotactic mechanism involved in the recruitment of peripheral immune cells to the paraventricular nucleus of the hypothalamus (PVH) upon inflammatory stimuli [107]. These authors showed that blockage of this recruitment by the peripheral administration of a CCR2 antagonist results in a reduction in local inflammation, suggesting that the MCP-1/CCR2 axis may also be involved in the chemotaxis of peripheral myeloid cells seen in HFD-induced hypothalamic inflammation. Curiously, according to the scRNA-seq data from ARC and ME published by Campbell et al. [108], MCP-1 expression in these areas is mainly observed in microglia. Hence, upon high-fat feeding, neurons could be the main cells involved in fractalkine production, while astrocytes and microglia are responsible for CCL2 (MCP-1) release. Based on these data, activated astrocytes not only directly modulate microglial activity, through the increased synthesis of pro-inflammatory cytokines and growth factors, but also mediate and stimulate the recruitment of peripheral macrophages to the hypothalamus, thus contributing to sustained microglial activation.

A leaky BBB in the hypothalamic area has been observed in the HFD-induced inflammatory response [109]. The mechanism underlying this BBB disruption may be due to various factors, such as altered function/structure of non-fenestrated brain endothelial cells, tanycytes, pericytes, neurons, or glia [11]. These cells constitute the neurovascular unit and confer integrity to the BBB under physiological conditions [110]. However, in response to some inflammatory stimuli, astrocytes and tanycytes increase the secretion of vascular endothelial growth factor (VEGF), increasing BBB permeability [110,111]. It is not well established whether this mechanism is crucial for the entrance of FFAs and peripheral cells into the hypothalamic parenchyma upon chronic HFD intake. Therefore, Lee et al. [12] showed that PVMs in the hypothalamic area produce inducible nitric oxide synthase (iNOS) abundantly under high-fat feeding, resulting in disruption of BBB integrity and in the spread of monocyte-derived macrophages in the ARC. As expected, specific hypothalamic macrophage iNOS inhibition completely abrogates astrocytic lipid droplets and macrophage accumulation and activation in the ARC of obese mice.

Another possible mechanism by which microglia and astrocytes contribute to the leaky BBB – observed in several inflammatory diseases – is the downregulation of tight-junction proteins, namely claudin-5 (CLDN5), occludin, and zonula occludens-1, in activated glial cells [112,113]. Interestingly, under systemic inflammation, vessel-associated microglia are able to phagocytize astrocytic end-feet, an action that damages BBB function [114]. However, how hypothalamic microglial and astrocytic crosstalk impairs BBB integrity under HFD-induced inflammation has been minimally explored.

Unsurprising, upon hypothalamic inflammation, microglial activity can also modulate astrocytic functions. Studies using co-culture of microglia and astrocytes have shown that microglial production of pro-inflammatory cytokines increases astrocytic glucose uptake, thus reducing intercellular glucose trafficking [115] and inhibiting astrocytic gap junctions [116,117]. Phenotypic alterations observed both in microglia and astrocytes at the onset of the inflammatory response are mediated by metabolic changes, switching from mitochondrial oxidative phosphorylation to glycolysis [118]. An important mechanism involved in HFD-induced hypothalamic inflammation in rodents comprises the increased levels of microglial UCP2 levels after HFD consumption, which drives a disruption in microglial mitochondrial dynamics [119]. These authors found that UCP2 deletion specifically in microglia reduced the number of GFAP+ cells in the ARC of mice fed a HFD, suggesting that mitochondrial dynamics in microglia regulate astrogliosis in HFD-induced hypothalamic inflammation.

**Figure 2:**
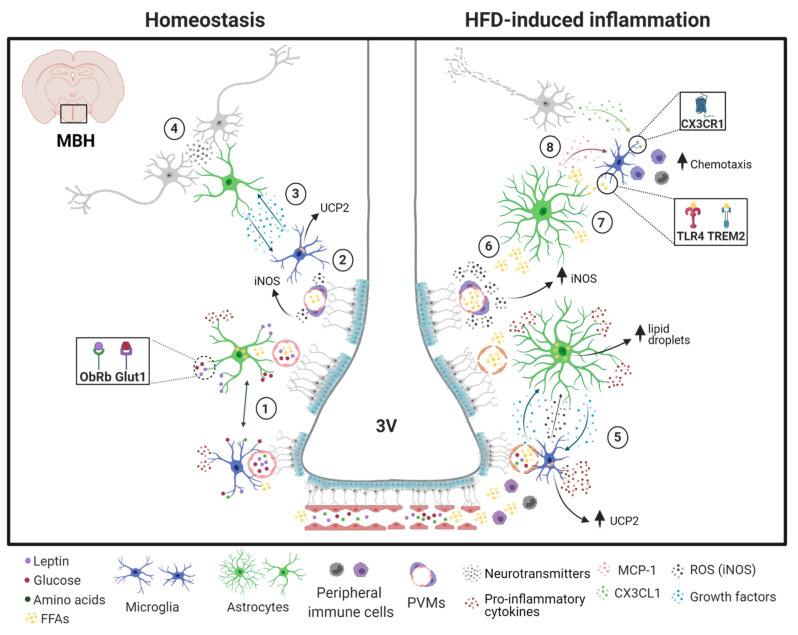
Microglia and astrocyte crosstalk in the hypothalamus. In homeostatic conditions: (**1**) microglia and astrocytes sense nutrients (amino acids, glucose, and free fatty acids [FFAs], among others) and hormones (e.g., leptin) that enter the hypothalamic parenchyma and react to those environmental changes. Astrocytes regulate lipid metabolism and storage and trigger metabolic changes due to hormone binding to their surface receptors (e.g., leptin and glucose receptors). (**2**) UCP2 expression in microglia is tightly controlled preserving their mitochondrial function while PVMs release normal levels of iNOS in response to FFAs concentration in blood vessels. (**3**) Microglia and astrocytes interact via contact-dependent and secreted factors and (**4**) control synapse development and function. Under high-fat feeding: (**5**) Glial cells quickly sense environmental changes and become reactive, releasing several pro-inflammatory cytokines and growth factors (e.g., VEGF and TGF-β1), and increasing reactive oxygen species (ROS) production. Astrocytes store the excessive FFAs in lipid droplets while microglial UCP2 increases, promoting astrogliosis and mitochondrial dysfunction in microglia. (**6**) PVMs surrounding blood vessels increase the release of iNOS promoting leaking BBB and facilitating the entrance of FFAs into the hypothalamic parenchyma, (**7**) which can stimulate IKKβ/NF-κB signaling by activating TLR4 and TREM2 in microglial cells. (**8**) Glial cells and neurons increase the synthesis of chemokines (e.g., MCP-1 and fractalkine), leading to the recruitment of peripheral myeloid cells to the central nervous system. If the inflammatory stimulus persists, the astrocytic control of synaptic functions become impaired, resulting in neuronal dysfunction. Abbreviations: 3V, third ventricle; MBH, mediobasal hypothalamus.

The schematic representation of the crosstalk between microglia and astrocytes under homeostasis and HFD-induced hypothalamic inflammation is shown in Figure 2.

Over the past decade, accumulating evidence has demonstrated that astrocytes also display high heterogeneity. These cells present many subpopulations spread throughout the CNS, which can vary depending on age, species, and sex, and astrocytic state (surveillance or reactive) [120,121,122]. Given their complexity, a consensus statement about reactive astrocytes [123] recently recommended that astrocyte phenotypes should be defined by a combination of molecular markers and functional readouts. There is evidence that heterogeneity of astrocytes and microglial hallmarks expression can be bi-directionally controlled [124]. Although understanding how different glial subpopulations regulate their local niche under HFD-induced hypothalamic inflammation still need to be investigated. Future studies regarding single-cell transcriptomic heterogeneity of hypothalamic astrocytes and microglia will elucidate which of their sub-populations are paired, their features, and how their hallmarks change upon HFD-induced reactivity.

## 4. Advances in Experimental Manipulation of Microglia

The identification of the microglial transcriptomic signature and heterogeneity has given rise to the development of new experimental models for manipulating or labelling specific subtypes of microglia. The hypothalamus has many microglial subpopulations that distinctly react to hormones and nutrients from the diet. Thus, the availability of these new models can provide a significant advance to comprehend the involvement of each microglial subtype in the HFD-induced hypothalamic inflammation and other inflammatory or neurodegenerative diseases. To achieve that, researchers should be able to choose the best model to answer their specific questions. In this topic, we discuss the newest approaches that have been developed.

### 4.1. Ex Vivo Models

To understand how microglia react to different stimuli, most studies conducted in the past decades have employed ex vivo strategies. A large variety of mouse microglia cell lines have been generated over the years, but the most used has been the murine microglial BV2 cell line. These cells were originally obtained and immortalized from the cerebral cortex of neonatal mice [125]. Other similar cell lines have been obtained from the whole brain, cerebellum, or cortex of adult/embryo rodents and have been less applied in basic research and barely cited in the literature compared with BV2 [126].

Most studies investigating microglial hypothalamic features using ex vivo methods have been conducted with BV2 cells [127,128,129]. It is well known that there are many phenotypic and genomic differences between microglia from the cortex and from the hypothalamus or microglia from a neonatal mouse compared to an adult or embryo – not to mention the fact that these cells were immortalized 30 years ago, and have very likely already suffered several transcriptomic modifications since that time. Another concern that must be considered when interpreting data is whether these cells are at the surveillance or reactive state when cultivated with no other neuronal or glial cells. Many questions and worries emerged when microglia stopped being seen as a simple macrophage of the CNS. Hence, to reduce any possible bias, published data and ongoing experiments with BV2 cells should be carefully planned, analyzed, and always accompanied by other in vivo strategies.

**Figure 3:**
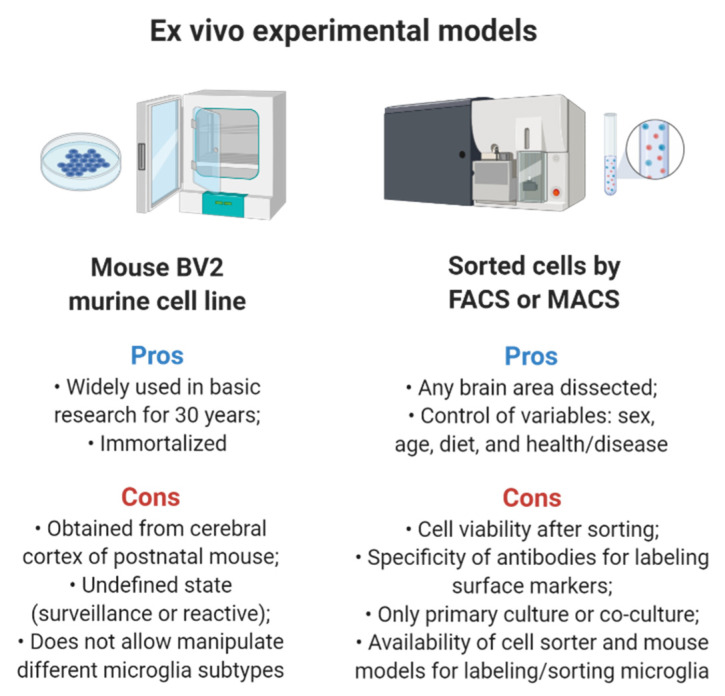
Comparisons between ex vivo experimental models for studying cultured microglia. Pros (in blue) and Cons (in red) of each approach are depicted in figure. Abbreviations: FACS, fluorescence-associated cell sorting; MACS, magnetic-activated cell sorting.

The development of new transgenic mouse models that express a fluorescent protein driven by microglial hallmarks promises an appealing alternative for researchers interested in cultured microglia. Magnetic-activated cell sorting (MACS) [130] and fluorescence-activated cell sorting (FACS) [131,132] using these fluorescent reporter mice or even microglia-specific surface markers allow the physical isolation of various subsets of microglia, which can be obtained from primary cultures or co-cultures with other cells. Using these approaches, the brain area of interest can be precisely harvested after a specific dietary or treatment protocol, and the researcher can freely choose the age and the sex of the rodents used in the experiments. These strategies render the collected data much more reliable when compared to studies using only BV2 cells. Although, when choosing cell sorter techniques researchers should keep in mind that any steps of protocols can trigger some reaction in microglia morphology or gene expression and even impair the cell viability (Figure 3).

Grassivaro et al. [133] recently applied FACS to isolate CNS resident microglia and peripheral myeloid cells from some models of neuroinflammation, such as experimental autoimmune encephalomyelitis (EAE). These authors collected samples during embryonic and postnatal periods and subsequently targeted cells from the brain and liver through labelling microglia and peripheral myeloid cells with fluorescent antibodies. By this approach, they observed extraordinary differences and transcriptomic details between those cells. Unfortunately, there is still a lack of data obtained through FACS or MACS for isolating microglia from the hypothalamus of experimental models of obesity.

### 4.2. In Vivo Models

Most studies need to evaluate microglial functions in vivo. Conveniently, at least in rodents, functional contributions of specific cell populations can be explored using Cre recombinase–mediated mutagenesis. The identification and cloning of CX3CR1 triggered the development of new tools for studying microglia in vivo [134,135,136]. Studies regarding the mechanisms through microglia-mediated HFD-induced hypothalamic inflammation have been mostly conducted with a transgenic mouse known as CX3CR1-Cre, which expresses Cre recombinase under the direction of the *Cx3cr1* promoter in the mononuclear phagocyte system, and CX3CR1^EGFP^ knock-in/knock-out reporter mice, which expresses a green fluorescent protein (EGFP) in monocytes, dendritic cells, natural killer cells, and brain microglia under the control of the same locus [20,137]. Consequently, all mechanisms regarding microglial reactivity and gliosis in those research articles can only be interpreted as adaptations and features of CX3CR1+ microglia. Thus, when researchers activate/inhibit microglia in CX3CR1-Cre mice through DREADD, optogenetic, or other Cre recombinase-targeted manipulation, they can only interpret the obtained data in the context of the manipulation of CX3CR1+ microglial, and not to other cells.

CX3XR1-Cre^ERT2^ is another *Cx3xr1*-driven model widely used in experimental research since it expresses Cre recombinase in a tamoxifen-inducible manner [138,139,140]. This tamoxifen-inducible model allows the temporal manipulation of microglia. Therefore, Van Hove et al. [141] have recently warned that the use of this strain is not indicated for all types of microglia-related investigations, such as fate-mapping studies, once it exhibits considerable leakiness in the absence of tamoxifen.

For many years, *Cx3cr1*-driven lines were largely employed in basic research – and they still are. However, they are haploinsufficient for *Cx3cr1*, which means that EGFP or Cre coding regions have been designed to replace the endogenous *Cx3cr1* locus. Some studies suggest that this haploinsufficiency could affect microglial functions, such as synaptic plasticity [142,143,144]. Given this background, all Cx3cr1-drive lines should be used with this limitation in mind. It is fortunate that new transgenic mouse models for genetic labelling and manipulating other microglial subtypes, myeloid cells and border-associated macrophages (BAMs) found in the hypothalamus and in other brain areas have been generated in recent years, allowing a deep investigation of new microglial phenotypes and functions (Table 1).

**Figure 4:**
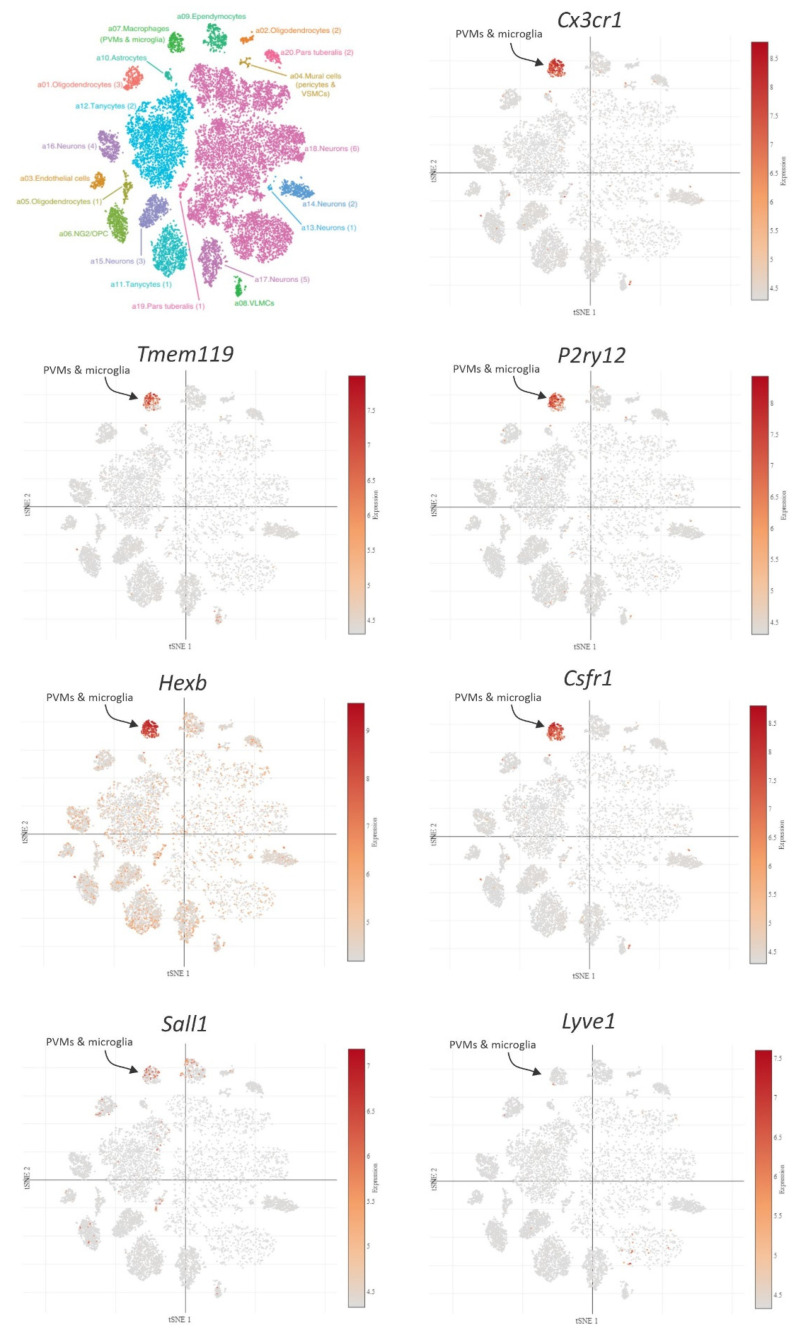
Distribution of genes in the arcuate nucleus (ARC) and median eminence (ME) chosen to generate mouse models that target microglia and perivascular macrophages (PVMs). Scatter plot images were obtained from https://singlecell.broadinstitute.org/ accessed on 20 November 2020, ARC-ME DROPSEQ data is from Campbell et al. [108].

Masuda et al. [150] recently developed another of these new microglia gene-targeting models. For this endeavor, the authors initially analyzed microglia and CNS-associated macrophage (CAM) signatures obtained from scRNA-seq, during homeostasis and disease, and identified beta-hexosaminidase subunit beta (*Hexb*) as a stably expressed microglia core gene. Based on that, they generated Hexb^TdTomato^ and Hexb-Cre^ERT2^ mouse strains, which express a red fluorescent protein (TdTomato) or Cre recombinase protein under the control of the *Hexb* locus upon tamoxifen administration, respectively. Although this research article was published a few months ago, there is no data available in the literature about the participation of microglial cells Hexb+ in hypothalamic inflammation. Curiously, *Hexb* is highly expressed in the ARC and ME [108], but in these areas, it is not tightly restricted to microglial when compared with other markers that can also be manipulated through transgenic mouse strains (*Cx3cr1*, *P2ry12*, or *Tmem119*) (Figure 4). Nevertheless, future studies should evaluate whether these Hexb+ microglia are important in some stage of HFD-induced hypothalamic inflammation and characterize their interactions with other glial and non-glial cells in the hypothalamus.

Transmembrane protein 119 (*Tmem119*) and purinergic receptor P2Y12 (*P2ry12*) loci have also been targets for the development of new mouse models (Table 1). Kaiser and Feng [147] generated Tmem119^EGFP^ mice and Tmem119-Cre^ERT2^ mice, which express EGFP or Cre^ERT2^ under the control of the Tmem119 coding region, respectively. More recently, Ruan et al. [149] generated another reporter mouse, Tmem119^TdTomato^, which expresses TdTomato rather than EGFP in microglia. All these Tmem119-driven lines have been validated and are now valuable tools to study specifically the role of Tmem119+ microglia in health and disease. McKinsey et al. [148] recently published the new P2ry12-Cre^ERT2^ mouse line. As stated by the authors, they chose P2ry12 because it appeared to be the most restricted to brain myeloid cells compared with other markers. Following the pattern, this model expresses Cre protein recombinase under the control of the P2ry12 locus. They also suggested that this model could be useful for studies about middle cerebral artery occlusion–induced ischemic stroke as well as EAE.

As previously mentioned, Valdearcos et al. [20] identified that both Tmem119+ and P2ry12+ cells react differently to Cx3cr1+ or Iba1+ cells in the ARC and the VMH under prolonged HFD intake. Thus, these novel *Tmem119*- and *P2ry12*-driven strains can also be promising to comprehend the mechanisms through which these specific microglial subtypes react to SFAs from the diet in this specific inflammatory condition.

Beyond Cre-driven and fluorescent reporter mouse lines, many researchers have chosen to deplete microglia to evaluate their role in some pathologic condition. There are many microglial depletion strategies, which were recently remarkably reviewed by Miron and Priller [151], including the use of liposomes, global knockout of genes required for microglial development or survival, or even transgenic or pharmacological induction of microglial death. Unfortunately, all these methods have some bias and do not allow the depletion of only microglial cells, because BAMs and monocytes may also be targeted. The use of some knockout mice, such as the knockout for colony stimulating factor 1 receptor (*Csfr1*-KO), is also not a good choice, because these mice lack all CNS macrophages; they show serious brain abnormalities and usually die within weeks after birth [152].

Due this dilemma, a novel model has been developed in which microglia are depleted, but BAMs are widely preserved after the genomic deletion of a super enhancer in the colony stimulating factor 1 receptor (*Csf1r*) coding region (fms-intronic regulatory element; Csf1r-FIRE^Δ/Δ^) [145]. A few studies have employed microglial depletion to study microglial immune and metabolic functions in the hypothalamus [137,153], because depletion disrupts immune balance and energy homeostasis [154]. Fortunately, Csf1r is also expressed by microglia from the ARC and ME [108] (Figure 4). CSF1R inhibition through a pharmacologic inhibitor (PLX5622) can improve metabolic outcomes in middle-aged female mice [155]. Thus, investigating the role of Csfr1+ microglial cells in the hypothalamic inflammation, through this Csf1r-FIRE^Δ/Δ^ mouse model, could also provide new, important information regarding this subtype of microglia.

As previously described, PVMs are also involved in HFD-induced hypothalamic inflammation [12]. This study was conducted with transgenic mouse models targeting lysozyme M (LysM)-expressing myeloid cells. Although many experimental models have been developed in the past years, there are still some methodological challenges with regard to manipulating specifically microglia or other CAMs/BAMs, such as PVMs. Even the above-mentioned transgenic lines used to study several microglial subsets (Table 1) have the mutation driven by a locus common to microglia and PVMs, at least in the ARC and ME [108] (Figure 4).

Some studies using scRNA-seq have already analyzed these cells separately [71], through dissection of leptomeninges, the perivascular space and parenchyma, and the choroid plexus; however, experimental manipulation of microglia and other CAMs/BAMs independently remains a challenge. Fortunately, Kim et al. [146] have just developed a binary transgenic system involving complementation-competent NCre and CCre fragments. Their expression is driven by two distinct promoters: Sall1^ncre^:Cx3cr1^ccre^, which specifically targets parenchymal microglia, and Lyve1^ncre^: Cx3cr1^ccre^, which allows the ability to target various CAMs throughout the brain. These new models are very promising and were developed using CRISPR/Cas9 technology. According to the authors, a CCre cassette was inserted after the *Cx3cr1* gene, and Ncre partner transgenes were inserted into *Sall1* and *Lyve1* loci, respectively. In fact, *Lyve1* is scarcely expressed by microglia, at least in the ARC and ME [108] (Figure 4). Hence, both models may be useful to study separately the functions of microglia and CAMs in HFD-induced inflammation, because the knowledge about the role of each glial cell type in this specific condition still need to be better clarified.

## 5. Microglial Signature Changes Upon HFD-Induced Hypothalamic Inflammation

There are sufficient clues indicating that microglia present distinct activated signatures under inflammatory conditions. Sousa et al. [72] performed scRNA-seq to investigate the microglial profile in the brain of LPS-injected mice. They found that microglia isolated from these mice exhibit a downregulation of their homeostatic signature together with an upregulation of inflammatory genes. They obtained this data by excluding other CNS-resident immune cells and peripheral cells in the analysis.

To explore deeply how HFD-induced inflammation affects microglial transcriptomics, we have searched for transcriptomic data published from HFD-fed rodents at a single-cell resolution. As already mentioned, Campbell et al. [108] performed Drop-seq profiling on the ARC and ME from mice across different feeding conditions, including 1-week HFD (60% calories from fat). They found that microglia present high *P2ry12* and low *Mrc1* while PVMs show low *P2ry12* and high *Mcr1*. However, when comparing low fat diet–fed mice with HFD-fed mice, they found the transcripts from these clusters downregulated, but they did not describe which HFD-sensitive genes were modulated.

**Figure 5:**
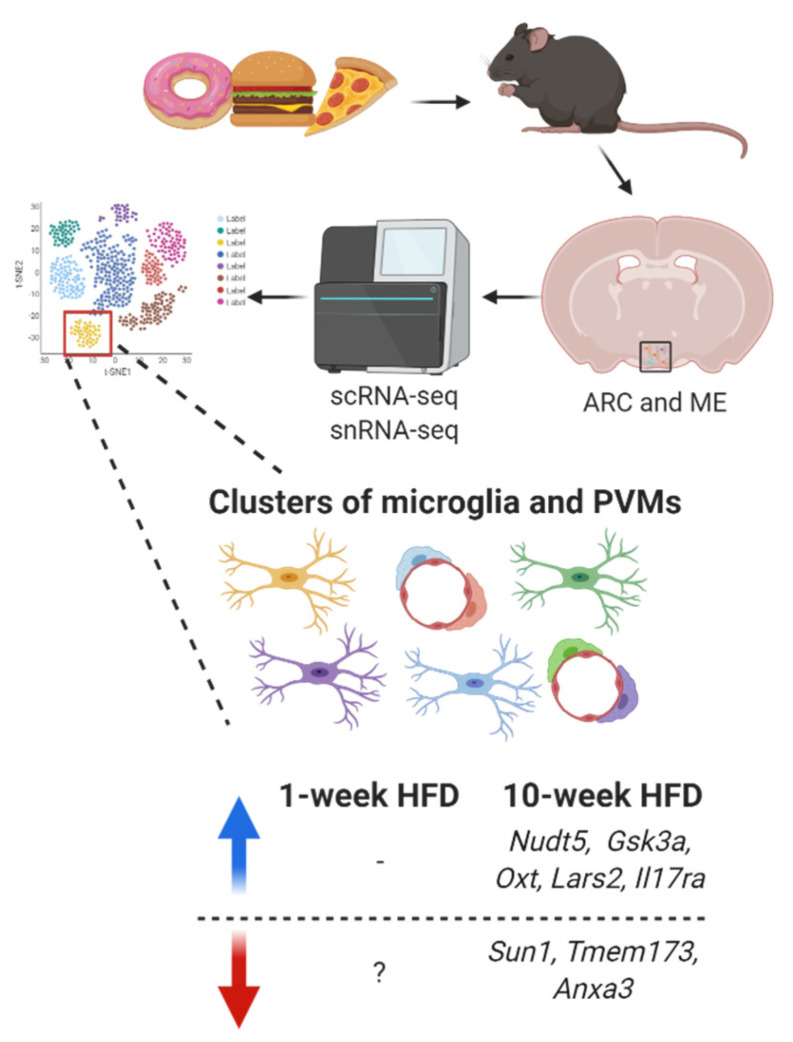
Transcriptomic signature changes in microglia (ramified cells) and perivascular macrophages (PVMs) (elongated cells around blood vessels) from the arcuate nucleus (ARC) and median eminence (ME) under short-term and prolonged HFD-induced hypothalamic inflammation. Blue arrow indicates upregulation while red arrow indicates downregulation.

On the other hand, in an experimental model of prolonged high-fat feeding, C57BL/6J male mice were fed a HFD (45% calories from fat) for 10 weeks, and their ARC was collected for single-nucleus RNA-seq (snRNA-seq) [156]. After consuming a HFD for 10-weeks, some genes were upregulated in the cluster of microglia (*Nudt5*, *Gsk3a*, *Oxt*, *Lars2*, and *Il17ra*) while other genes (*Sun1*, *Tmem173* and *Anxa3*) were downregulated (Figure 5).

Some of them have also been described in obesity-related studies. Methylation in *Lars2*, which is a mitochondrial gene, has been reported in an epigenome-wide association study as a gene associated with increased body mass index and waist circumference [157]. The receptor for interleukin-17 (IL-17), known as *Il17ra*, is found in pro-opiomelanocortin (POMC) and agouti-related peptide (AgRP) neurons in the hypothalamus and modulates food intake after IL-17 binding, without affecting whole-body energy expenditure [158].

Although the ARC is the main nucleus involved in energy metabolism and food intake control, functional impairments in neurons and glial cells from other hypothalamic nuclei are also involved in DIO establishment [159,160]. Recently, Rossi et al. [161] identified in the lateral hypothalamic area (LHA) thousands of genes altered upon prolonged HFD intake. This research article highlighted only dynamic transcriptomic details in neurons; thus, the authors did not deeply explore the transcription differences in microglia and other myeloid cell lineages upon. However, the authors distinctively represent these cells by using *Cx3cr1* hallmark for microglia and *Lys2* for myeloid cells, which was not considered in data from ARC published by Deng et al. [156].

Interestingly, from all transcriptomic data obtained from the ARC–ME or LHA, researchers can apply a bioinformatic analysis to better investigate how microglia and other myeloid cells changes upon prolonged HFD. In the face of the recent and continuous development of novel transcriptomic tools, such as scRNA-seq, snRNA-seq, and CyTOF, among others, future studies should be conducted to better clarify the main changes in microglial signature throughout the hypothalamus under different stages of high-fat feeding. For accurate data interpretation, microglia and other CNS-associated macrophages should be clustered and analyzed separately. The detailed identification of those cells will be valuable to answer more precisely several outstanding questions.

## 6. Conclusions

Microglia were first recognized as macrophage-like cells from the CNS a century ago, but for a long time their complexity was unknown. Luckily, the development of assorted transcriptomic tools has boosted the knowledge about these cells in recent years. Currently, it is well known that the hypothalamus presents several microglial subsets that can be identified by their hallmarks: *Iba1*, *Cx3Cr1*, *Tmem119*, *P2ry12*, *Trem2*, *Hexb*, and *Csfr1*, among many other. DM are also found in the hypothalamic area, but their roles in HFD-induced inflammation has been poorly investigated. In fact, microglia play a pivotal role in different stages of the hypothalamic inflammatory process, but how each microglial subtype reacts to SFAs from the diet, communicates with other cells, or even leads to the recruitment of peripheral myeloid cells remains to be explored. Beyond microglia, astrocytes also display high heterogeneity throughout the CNS, and in the hypothalamus, its diversity and its crosstalk with microglia require to be better elucidated. Novel experimental models for manipulating or labelling microglia have been developed and will be useful to answer that question in forthcoming research. On the other hand, the hypothalamic microglial signature under HFD-induced hypothalamic inflammation should still be further studied using different transcriptomic approaches. Together, these advances will allow researchers to take full advantage of crucial insights we have gained about microglial heterogeneity (provided by transcriptomic data) and to exploit this knowledge to determine the mechanisms by which microglia are involved in the inflammatory process observed in obesity.

## Figures and Tables

**Table 1:** Novel in vivo experimental models for studying various subtypes of microglia, myeloid cells, and BAMS.

Manipulating Microglia	Labeling Microglia	Depleting Microglia	Targeting Myeloid Cells	Targeting Microglia and PVMs
CX3CR1-Cre	CX3CR1^EGFP^	Csf1r-FIRE^Δ/Δ^ [145]	LysM^EGFP^	Sall1^ncre^:Cx3cr1^ccre^ [146]
CX3CR1-Cre^ERT2^	Tmem119^EGFP^ [147]		LysM-Cre	Lyve1^ncre^: Cx3cr1^ccre^ [146]
P2ry12-Cre^ERT2^ [148]	Tmem119^TdTomato^ [149]			
Tmem119-Cre^ERT2^ [147]	Hexb^TdTomato^ [150]			
Hexb-Cre^ERT2^ [150]				

## Data Availability

Not applicable.

## Abbreviations

ARC	Arcuate nucleus of the hypothalamus
BAMs	Border-associated macrophages
BBB	Blood–brain barrier
BMDC	Bone-marrow-derived cells
CAMs	CNS-associated macrophages
CCL2	Chemokine C-C motif ligand-2
CCR2	C-C chemokine receptor type 2
CD11b	Cluster of differentiation molecule 11B
CD169	Sialoadhesin
CNS	Central nervous system
CSF1R	Colony stimulating factor 1 receptor
CX3CR1	CX3C chemokine receptor 1
CyTOF	Mass cytometry
DAM	Disease-associated microglia
DIO	Diet-induced obesity
DM	Dark microglia
EAE	Experimental autoimmune encephalomyelitis
FACS	Fluorescence-activated cell sorting
FFAs	Free fatty acids
GFAP	Glial fibrillary acidic protein
Hexb	Beta-hexosaminidase subunit beta
HFD	High-fat diet
IBA1	Ionized calcium binding adaptor molecule 1
iNOS	Inducible nitric oxide synthase
ITGAM	Integrin subunit alpha M
LHA	Lateral hypothalamic area
LPS	Lipopolysaccharide
MACS	Magnetic-activated cell sorting
MCP-1	Monocyte chemoattractant protein-1
ME	Median eminence
P2ry12	Purinergic receptor P2Y12
PVH	Paraventricular nucleus of the hypothalamus
PVMs	Perivascular macrophages
ROS	Reactive oxygen species
scRNA-seq	Single-cell RNA sequencing
SFAs	Saturated fatty acids
Siglec	Sialic acid-binding immunoglobulin super-family lectin
snRNA-seq	Single-nucleus RNA sequencing
TGF-β	Transforming growth factor beta
TLR4	Toll-like receptor 4
Tmem119	Transmembrane protein 119
TREM2	Triggering receptor expressed on myeloid cells-2
VEGF	Vascular endothelial growth factor
VMH	Ventromedial nucleus of the hypothalamus
